# Supplementary material for: The allotetraploid origin and asymmetrical genome evolution of the common carp Cyprinus carpio
Source: Nat Commun. 2019 Oct 11;10:4625. doi: 10.1038/s41467-019-12644-1 (PMC6789147; doi:10.1038/s41467-019-12644-1)
Supplement: Supplementary file 1 — Supplementary Information [file 41467_2019_12644_MOESM1_ESM.pdf]

The allotetraploid origin and asymmetrical genome evolution of common carp

Cyprinus carpio

Xu et al.

SUPPLEMENTARY INFORMATION

TABLE OF CONTENTS

Supplementary Figures 1

Supplementary Figure 1. The photos of three sequenced common carp strains. 1

Supplementary Figure 2. High density genetic linkage maps of three subspecies of *C. carpio*. 2

Supplementary Figure 3. Comparison between GM and SP based on BAC-end sequences. 5

Supplementary Figure 4. Circos plot of three common carp genomes. 6

Supplementary Figure 5. Phylogenetic tree of closely related Cyprininae species based on *rag2* and mitochondrial genes. 9

Supplementary Figure 6. Sampling sites of the sequenced candidate diploid progenitors. 10

Supplementary Figure 7. Phylogenetic tree based on 2071 conserved homoeologous gene pairs. 11

Supplementary Figure 8. TE component in two subgenomes of common carp. 12

Supplementary Figure 9. Typical structure variations between homoeologous chromosomes of two subgenomes. 13

Supplementary Figure 10. Expression patterns of all expressed genes in the 12 tissues in *C. carpio*. 14

Supplementary Figure 11. Venn diagram of the 1,986 divergent gene pairs in *C. carpio* and genes from the three functional divergent clusters comparing *C. carpio and C. idella*. 15

Supplementary Figure 12. Clusters for 8291 homoeologous gene pairs in the 12 tissues in *C. carpio*. 16

Supplementary Figure 13. Expression patterns of *pdl1* and *acsl6* genes in 12 tissues in *C. carpio* 17

Supplementary Figure 14. Asymmetric homoeologous expression patterns under biotic or abiotic stresses in *C. carpio*. 18

Supplementary Figure 15. Methylation levels of asymmetrically expressed homoeologous genes in 12 tissues in *C. carpio*. 19

Supplementary Figure 16. CG methylation levels of divergent expressed homoeologous genes in *C. carpio*. 20

Supplementary Tables 21

Supplementary Table 1. Summaries of library construction and sequencing data of three common carp strains 21

Supplementary Table 2. The genome assembly statistics of three common carp strains. 22

Supplementary Table 3. Summaries of genetic linkage maps of three common carp strains. 23

Supplementary Table 4. Statistics of three integrated genome assemblies. 24

Supplementary Table 5. Completeness assessment of three common carp genomes. 25

Supplementary Table 6. Genic region coverage assessed based on ESTs in three common carp genome assemblies 26

Supplementary Table 7. Transposable elements in common carp genomes. 27

Supplementary Table 8. Gene prediction in common carp genome. 28

Supplementary Table 9. The statistics of gene structure of *C. carpio* and the comparison with other teleosts. 29

Supplementary Table 10. Gene annotation in common carp genome. 30

Supplementary Table 11. Accession numbers of *rag2* genes used in this study. 31

Supplementary Table 12. Genome sequencing and assembly of diploid species. 32

Supplementary Table 13. Chromosome nomenclature of *C. carpio* according to *D. rerio*. 33

Supplementary Table 14. Gene present and loss categories based on distinguished A/B subgenome. 34

Supplementary Table 15. Validation of homoeologous rearrangement using mate-paired BAC-end sequences. 35

Supplementary Table 16. Transcriptome data from 12 tissues of *C. carpio*. 36

Supplementary Table 17. Homoeologous gene expression divergence in two subgenomes. 37

Supplementary Table 18. Transcriptome data from 12 tissues of *C. idella*. 38

Supplementary Table 19. Homoeologous gene expression under abiotic and disease stresses. 39

Supplementary Table 20. Differentially expressed genes of 8291 homoeologous gene pairs under abiotic and biotic stress. 40

Supplementary Table 21. Differentially expressed homoeologous gene pairs of the 8291 homoeologous gene pairs under abiotic and biotic stress. 41

Supplementary Table 22. Whole genome methylation data of common carp. 42

Supplementary Table 23. Whole genome methylation levels of common carp.......43

Supplementary References...44

Supplementary Figures

Supplementary Figure 1. The photos of three sequenced common carp strains. Fish images are derived from the authors' laboratories.

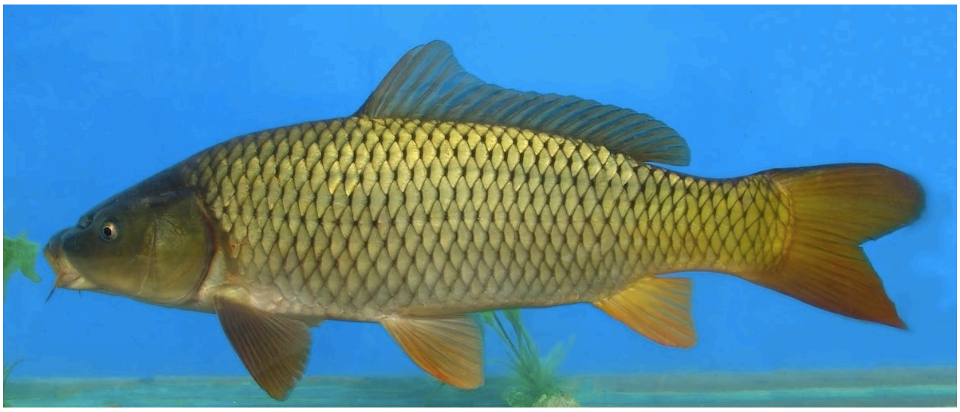

(A) Yellow River carp

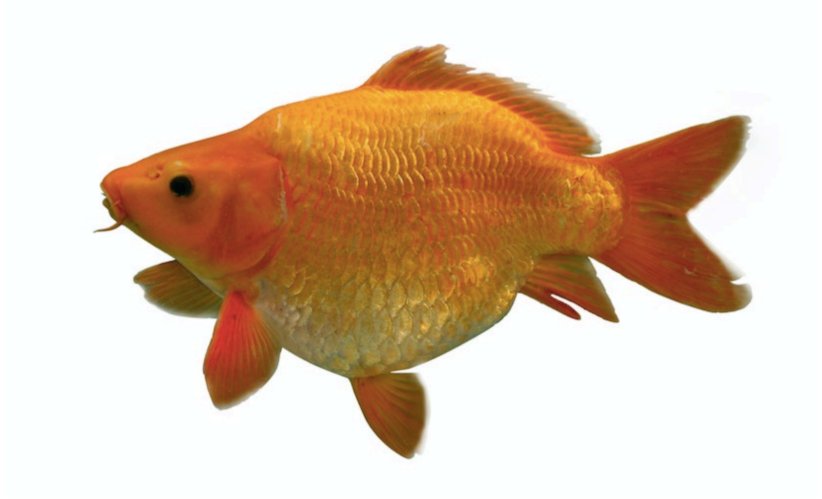

(B) Hebao red carp

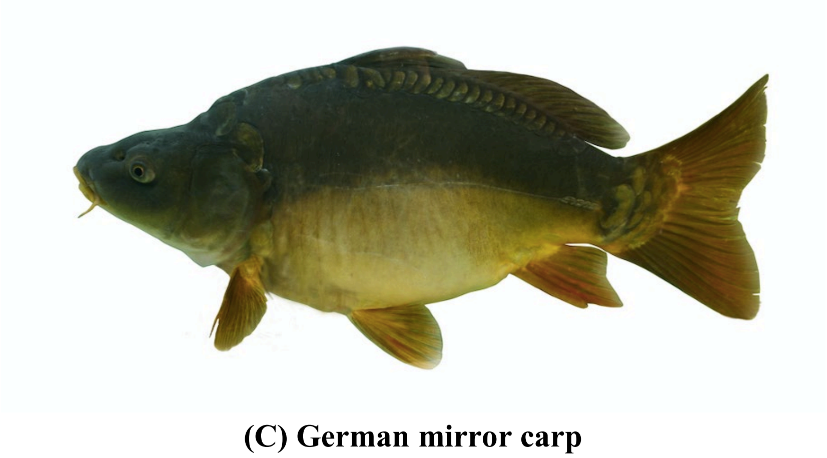

(C) German mirror carp

Supplementary Figure 2. High density genetic linkage maps of three subspecies of *C. carpio.*

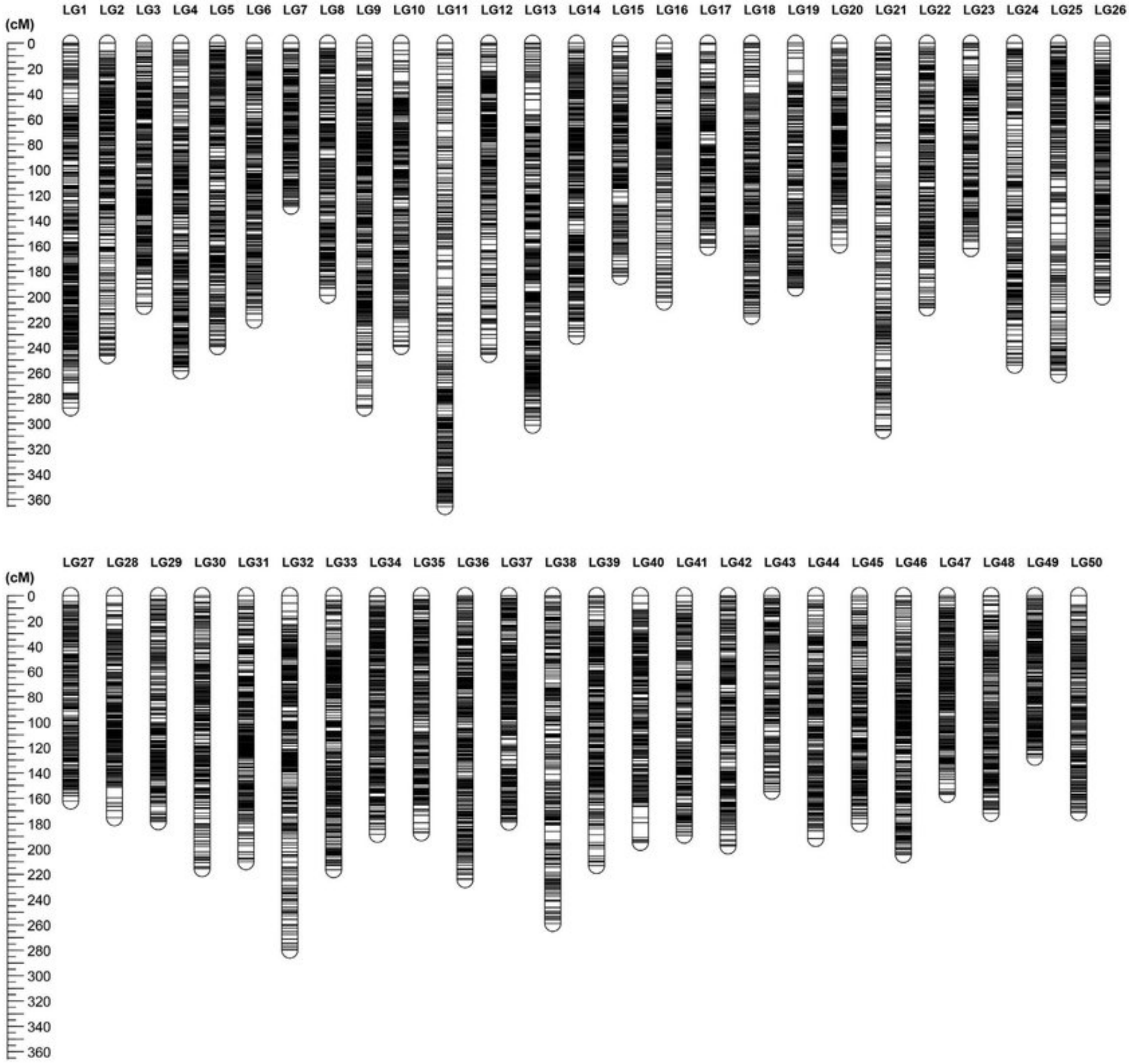

(A) Genetic linkage map of Yellow River carp

Note: The genetic linkage map for Yellow River carp has been previously published on Scientific Reports[1].

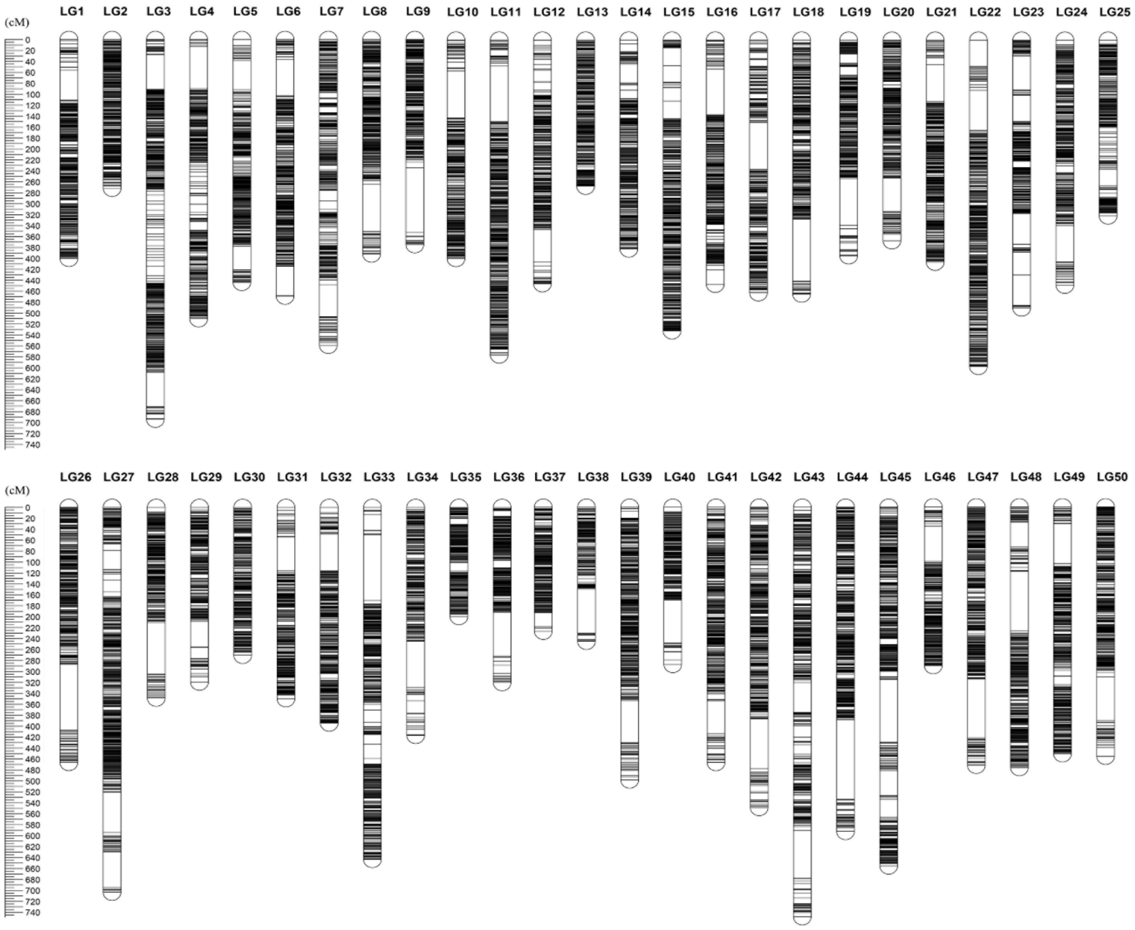

(B) Genetic linkage map of Hebao red carp

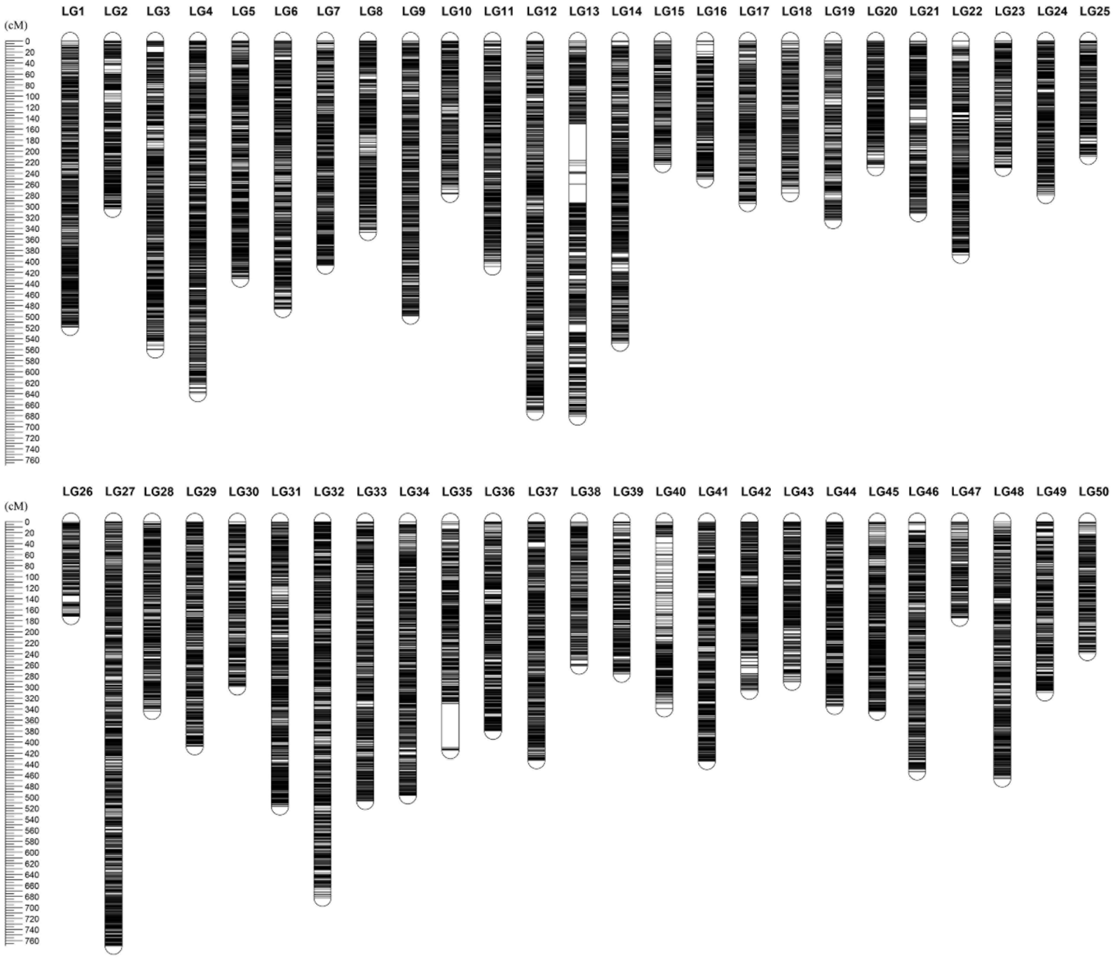

(C) Genetic linkage map of German mirror carp

Supplementary Figure 3. Comparison between GM and SP based on BAC-end sequences.

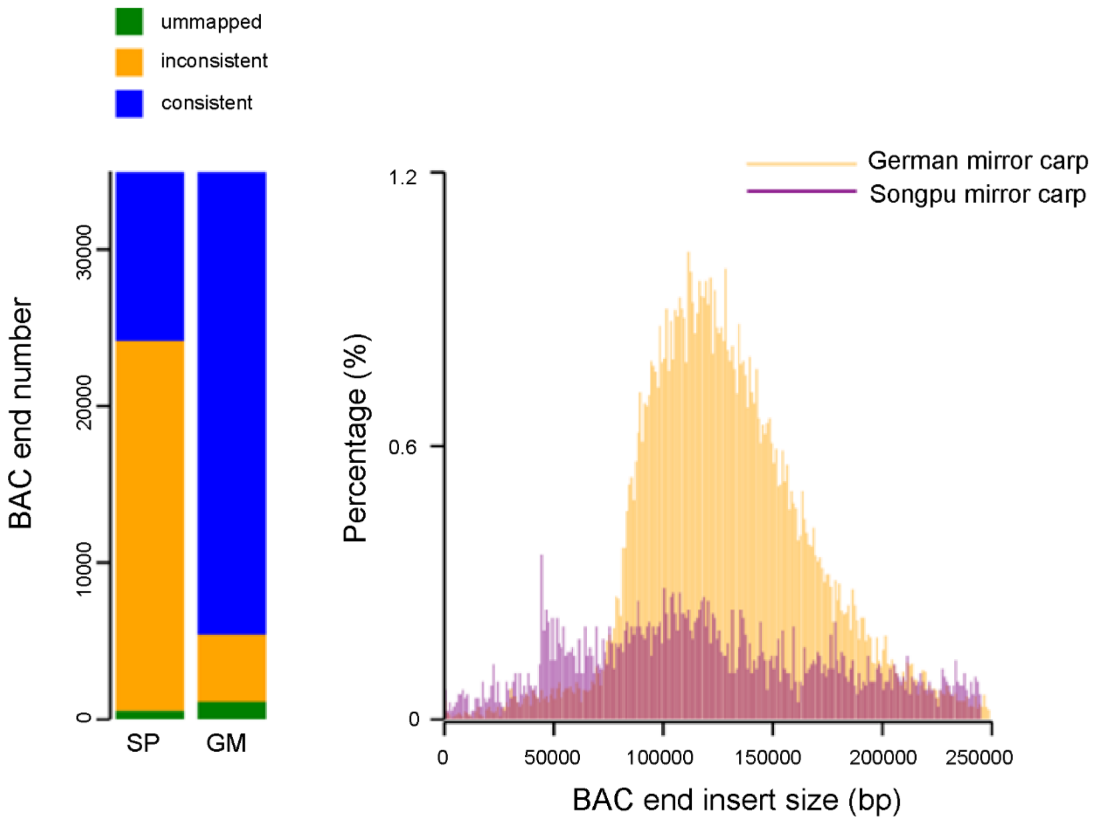

Supplementary Figure 4. Circos plot of three common carp genomes.

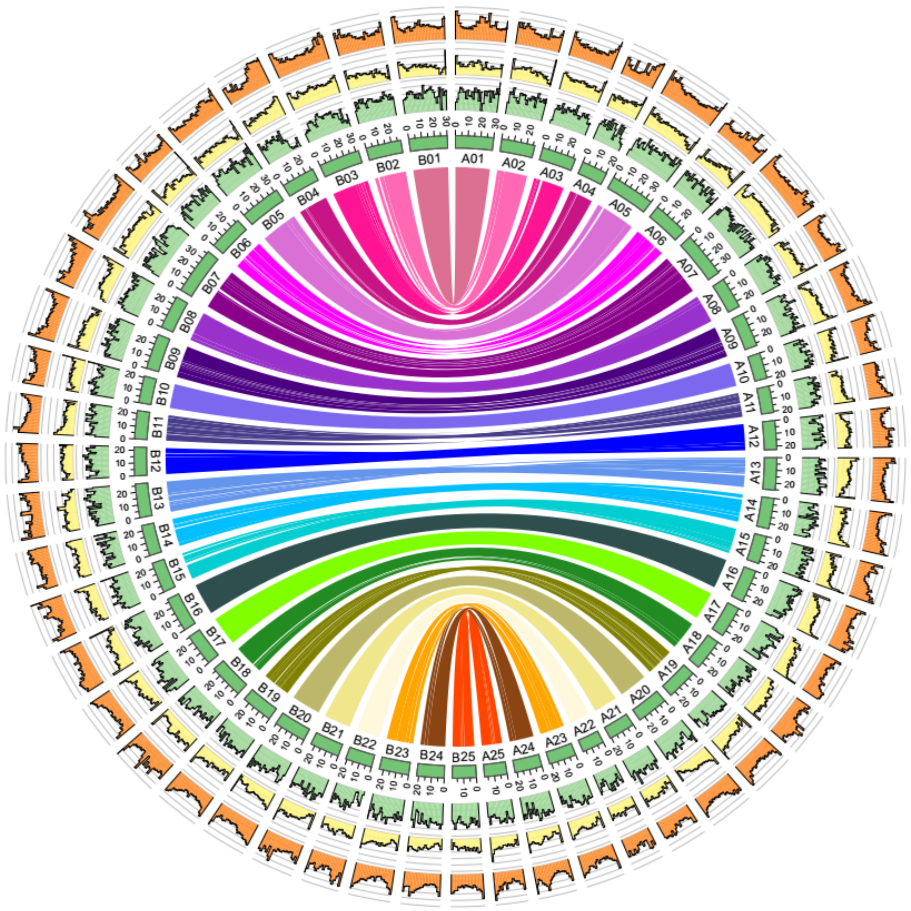

(A) Yellow River carp

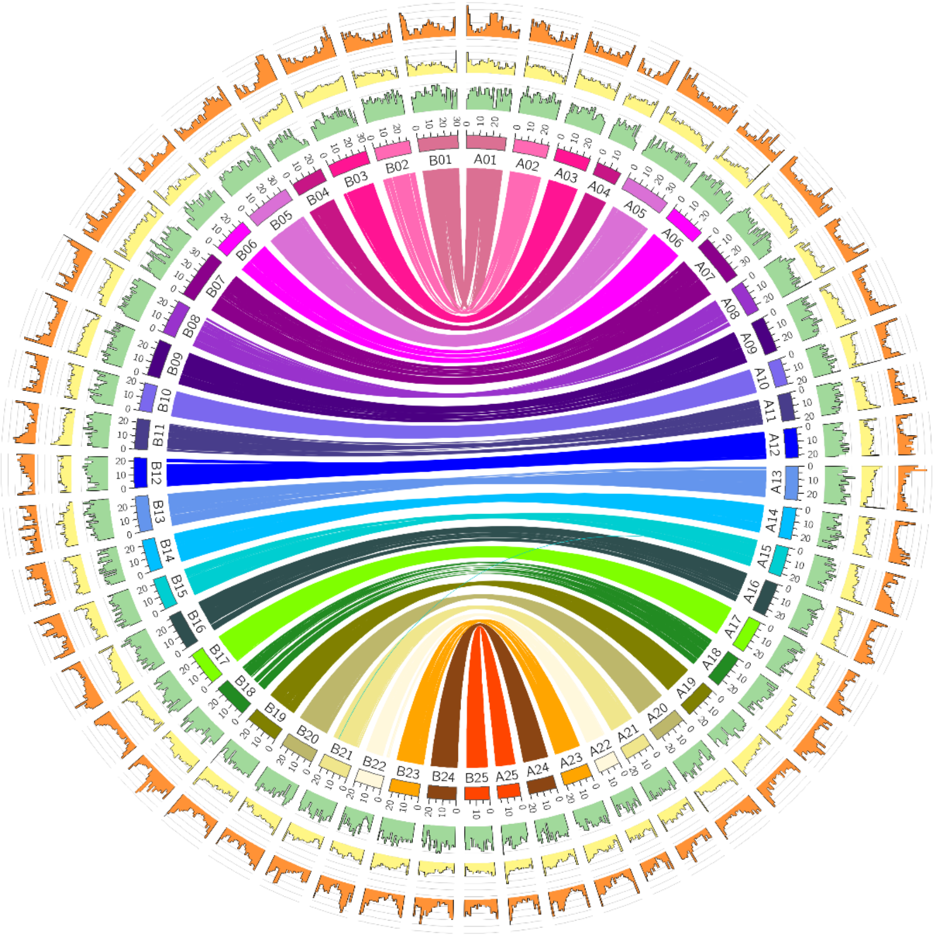

(B) Hebao red carp

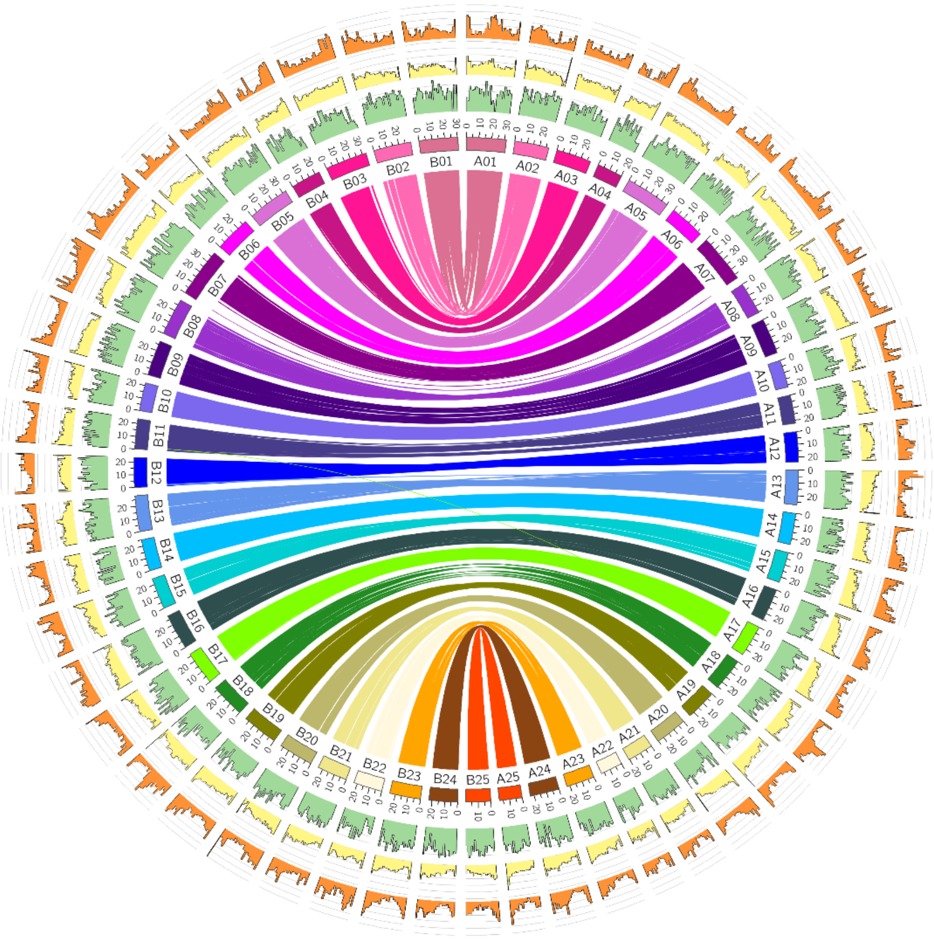

(C) German mirror carp

Note: Circos was used to plot repeat content within a 1-Mb sliding window (the outermost plot, orange), GC content within a 1-Mb sliding window (yellow), gene distribution on each chromosome within a 1-Mb sliding window (middle plot, green), assembled chromosomes and maps of the common carp A genome and B genome (innermost plot).

Supplementary Figure 5. Phylogenetic tree of closely related Cyprininae species based on *rag2* and mitochondrial genes.

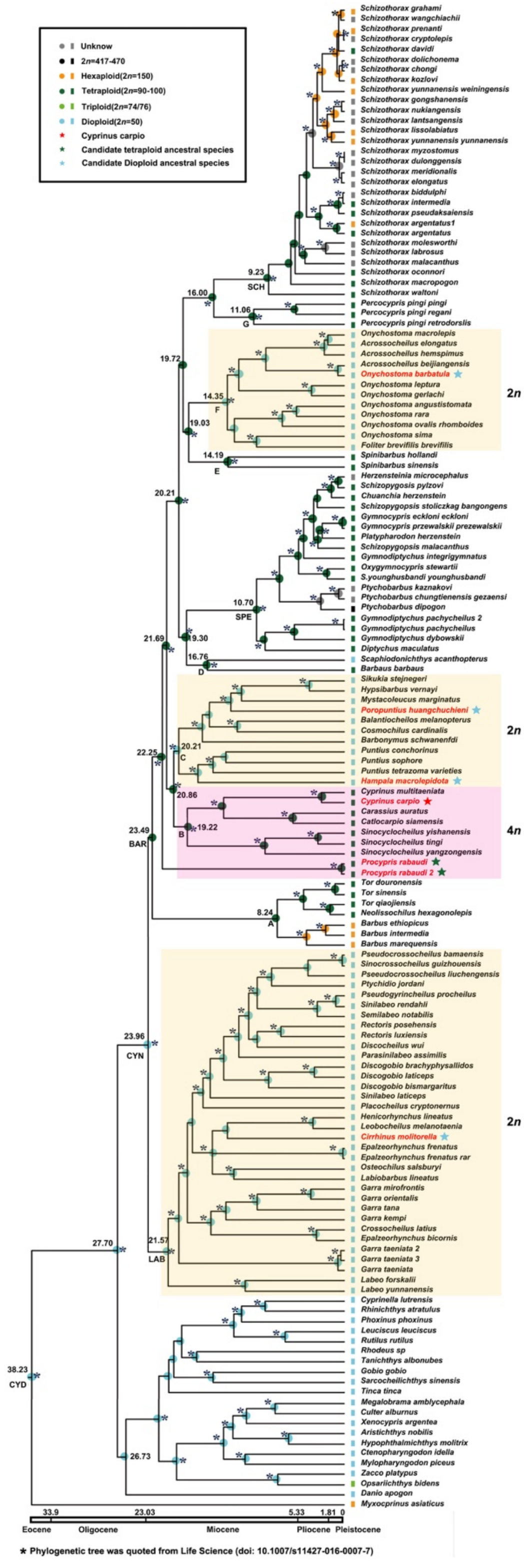

Note: Figure was modified based on Figure 2 from Wang, et al, 2016 [2].

Supplementary Figure 6. Sampling sites of the sequenced candidate diploid progenitors.

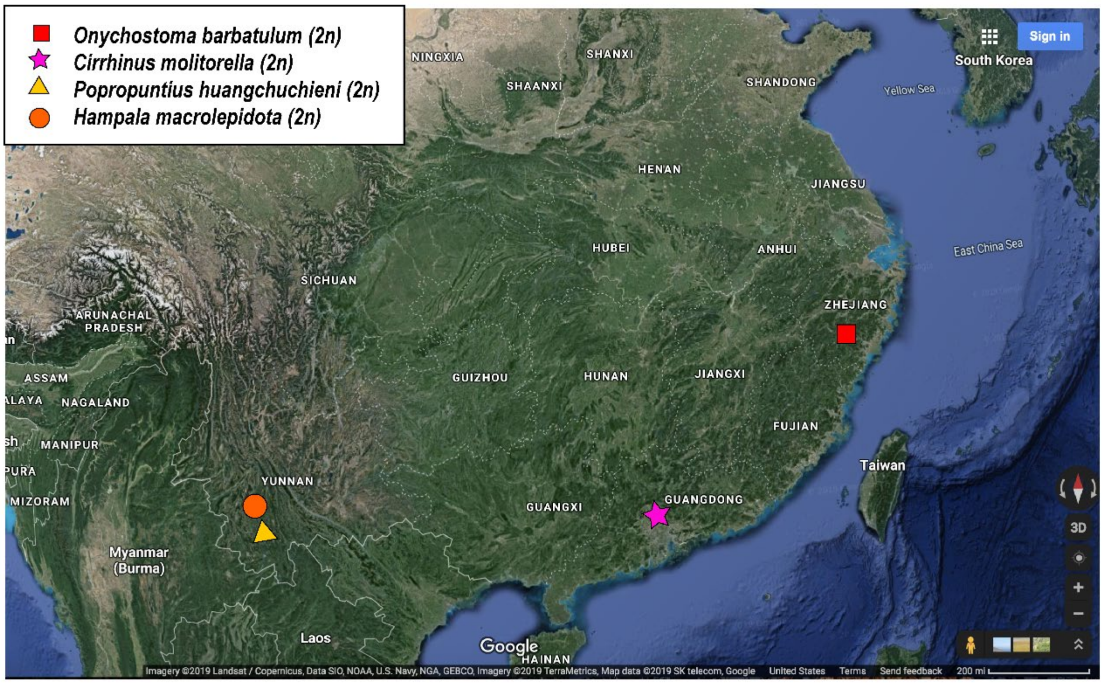

Popropuntius huangchuchieni and *Hampala macrolepidota* were collected at Xishuangbanna in Yunnan Province, China, *Onychostoma barbatulum* was collected at Lishui in Zhejiang Province, China, and *Cirrhinus molitorella* was collected at Guangzhou, Guangdong Province, China. Image data was retrieved from Google Map (http://maps.google.com).

Supplementary Figure 7. Phylogenetic tree based on 2071 conserved homoeologous gene pairs.

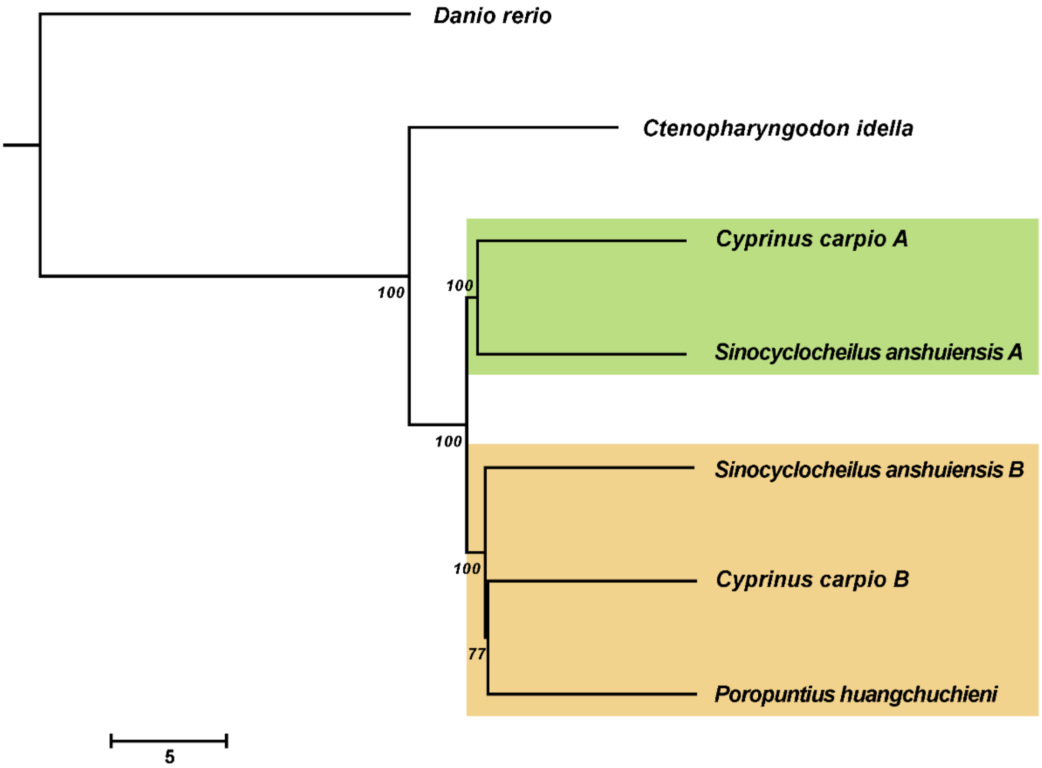

The 2,071 conserved homoeologous gene pairs were from two allotetraploids (*C. carpio* and *S. anshuiensis*) and their single-copy orthologs from three diploids (*D. rerio, C. idella* and *P. huangchuchieni*). The 2,071 phylogenetic trees were integrated using Bin-MPEST software.

Supplementary Figure 8. TE component in two subgenomes of common carp.

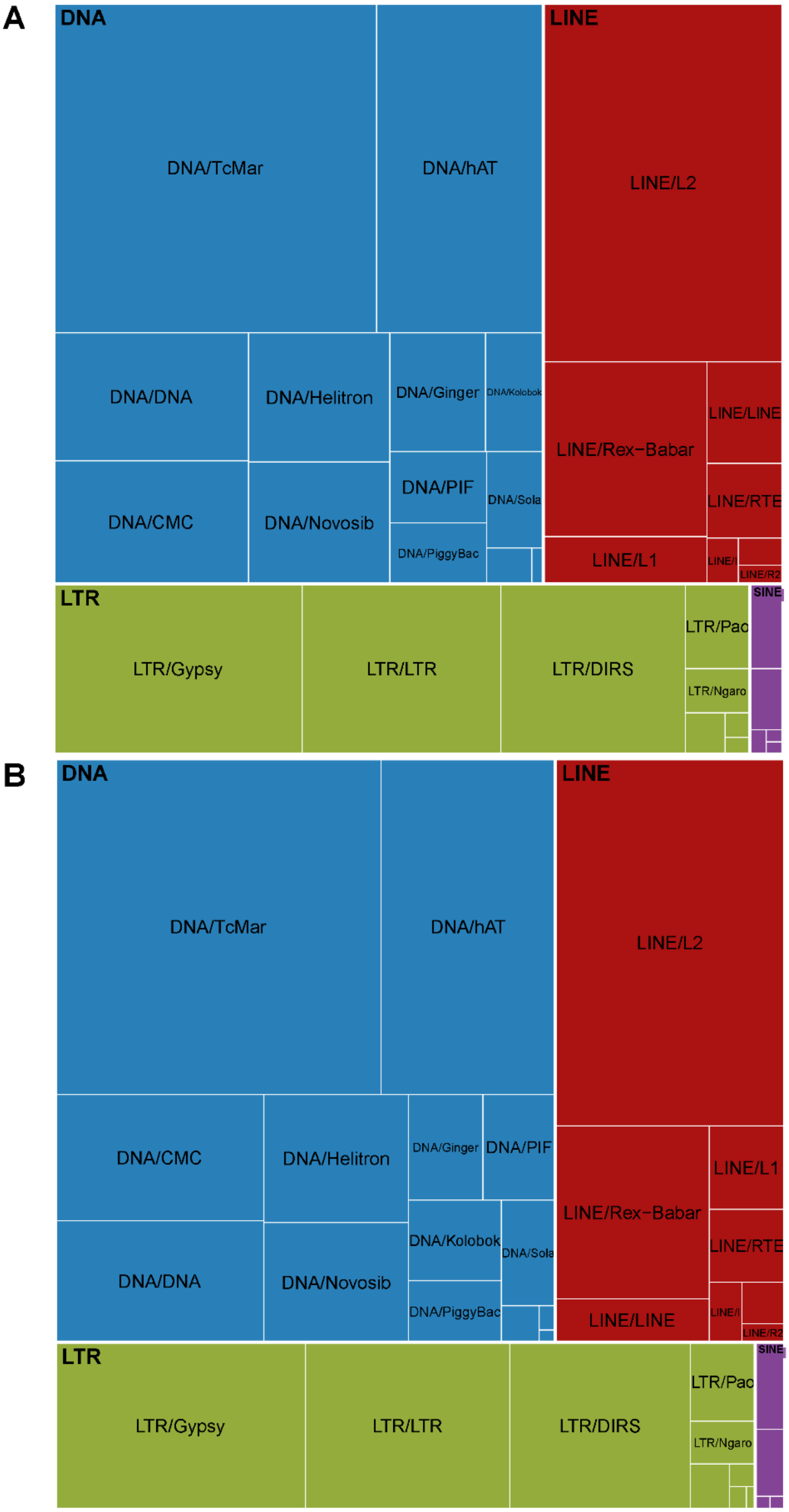

Note: **A**, subgenome A of common carp; **B**, subgenome B of common carp.

Supplementary Figure 9. Typical structure variations between homoeologous chromosomes of two subgenomes.

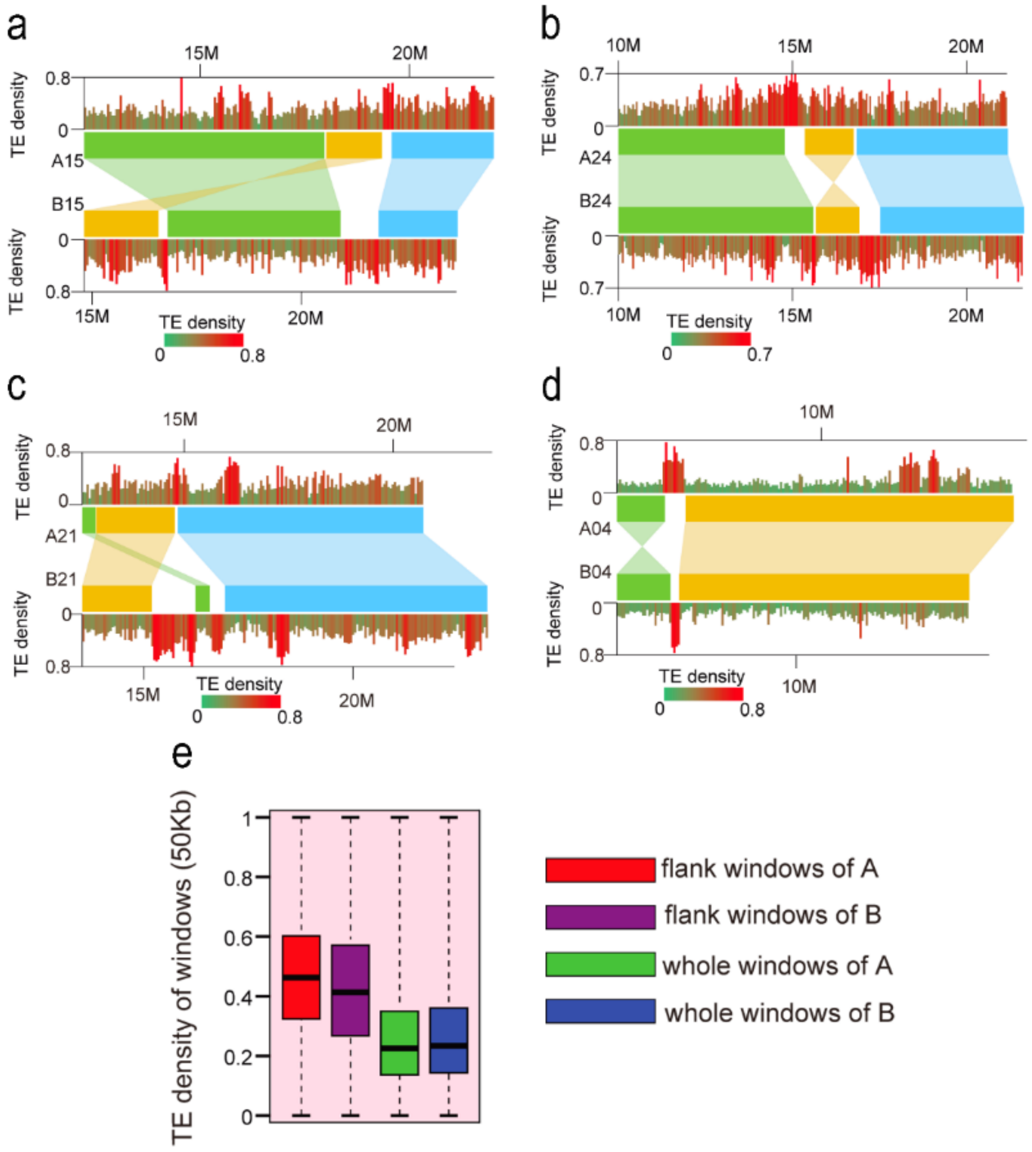

Note: Segmental translocation in the homoeologous chromosome A15/B15, A21/B21 (a,c) and segmental inversion in the homoeologous chromosome A24/B24, A04/B04 (b,d) of *C. carpio*; **e**, TE distribution in the flanking regions of the structure variations and chromosome-level TE distribution.

Supplementary Figure 10. Expression patterns of all expressed genes in the 12 tissues in *C. carpio*.

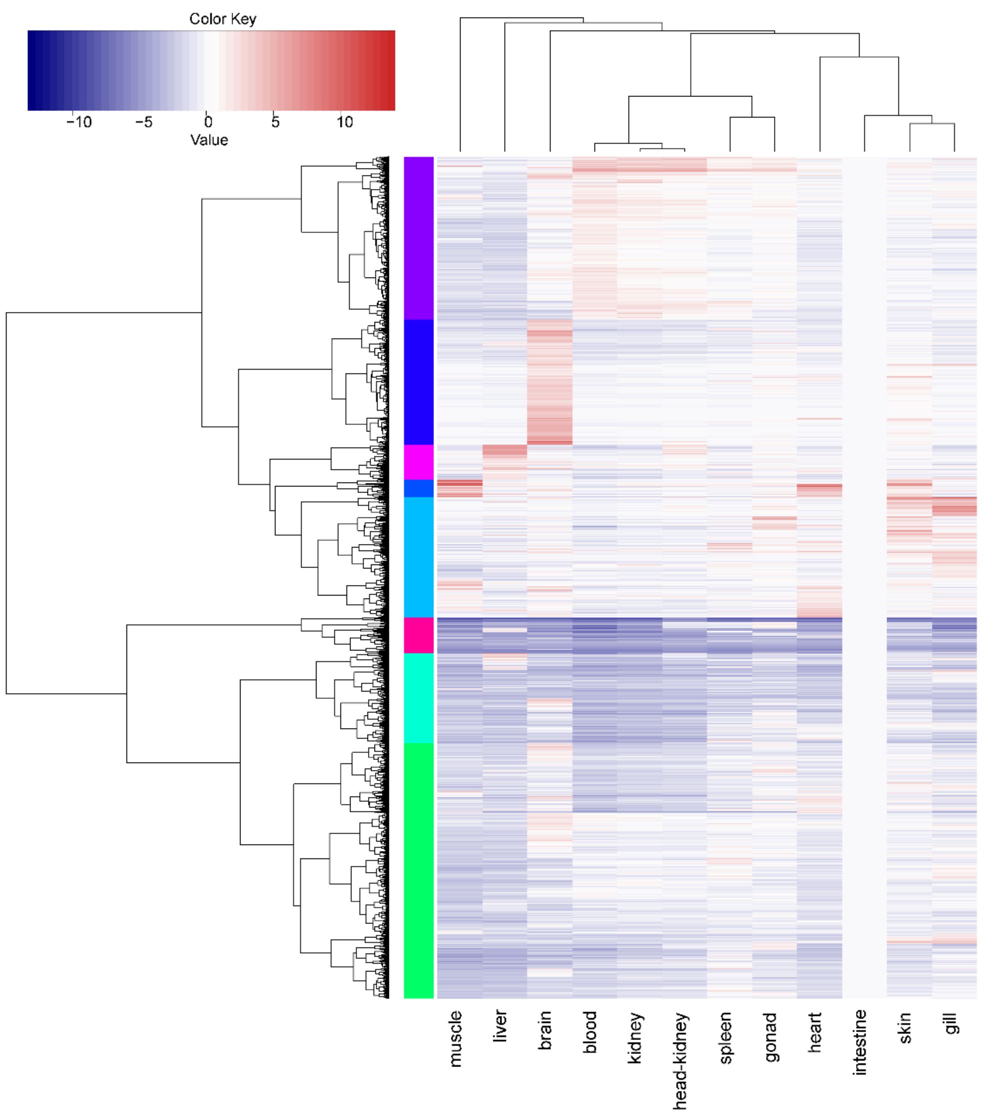

The genes with expression level greater than 1 in at least one tissue were expressed, and expression level of 24,000 genes were transformed to log2 (FPKM+1) for clustering in 12 tissues using Pearson's correlation and Ward's method in the R function hclust. The eight clusters were visualized as heatmaps using the R function heatmap (ggplot2). We identified a total of 1,986 homoeologous gene pairs with spatial expression divergence.

Supplementary Figure 11. Venn diagram of the 1,986 divergent gene pairs in *C. carpio* and genes from the three functional divergent clusters comparing *C. carpio and C. idella*.

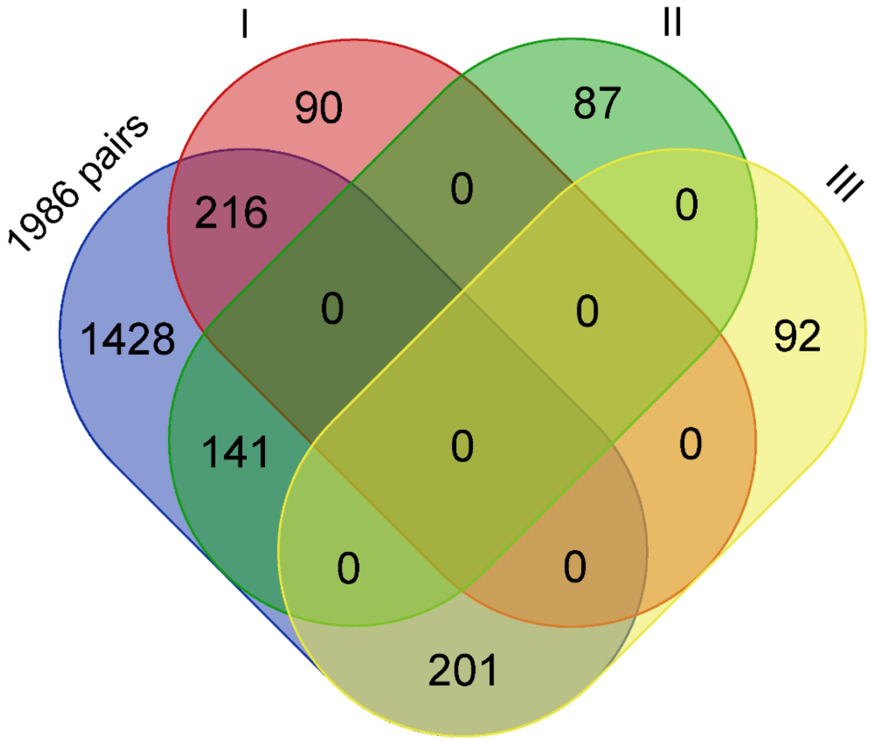

The 1,986 homoeologous gene pairs with spatial expression divergence were identified when built co-expression clusters in 8291 homoeologous gene pairs in two subgenomes in 12 tissues of *C. carpio* (Supplementary Figure 12). The three distinct sub-clusters was from the co-expression clusters in 8291 homoeologous gene pairs using expression values in 12 tissues of diploid grass carp *C. idella* as outgroup. A *C. carpio* gene was classified as conserved if the *P* value of Wilcoxon test to the *C. idella* orthologue was above 0.05 across the 12 common tissues, and diverged if the *P* value was below 0.05. 306 orthologous triplets were differentially expressed in *C. idella* and in two subgenomes of *C. carpio* (sub-cluster I); 228 orthologous triplets that have similar co-expression patterns in grass carp genome and subgenome B of common carp but different co-expression patterns in subgenome A (sub-cluster II); 293 orthologous triplets that have similar co-expression patterns in grass carp genome and subgenome A of common carp but different co-expression patterns in subgenome B (sub-cluster III). Majority (61.8%-70.6%)of the genes of three sub-clusters in Figure 3d were included in 1986 homoeologous genes.

Supplementary Figure 12. Clusters for 8291 homoeologous gene pairs in the 12 tissues in *C. carpio*.

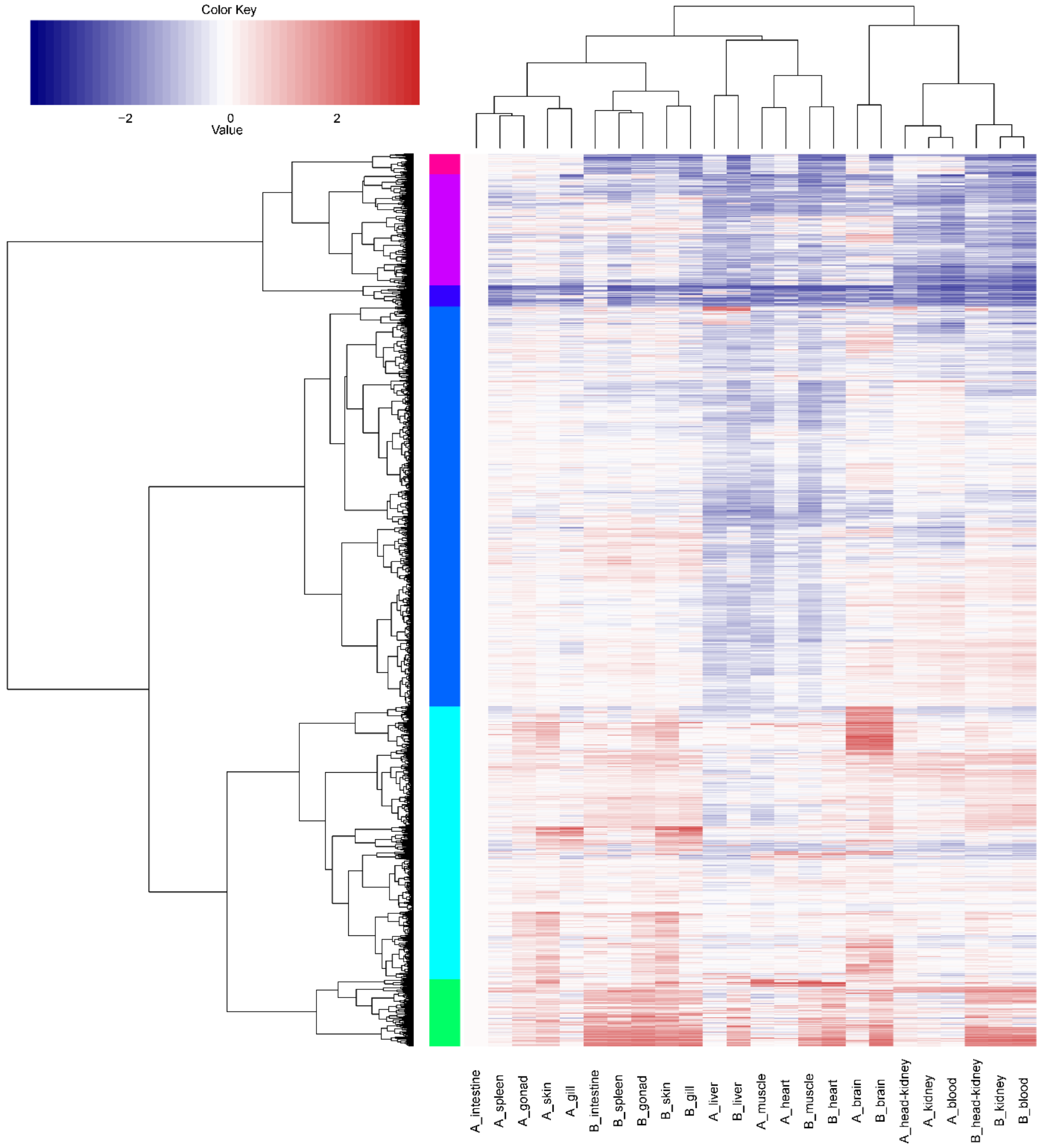

The expression levels of 8,291 homoeologous genes were clustered in 12 tissues in the two subgenomes using Pearson's correlation and Ward's method in the R function hclust. The six clusters were visualized as heatmaps using the R function heatmap (ggplot2), including the two highly divergent clusters in **figure 4c**, with 191 and 620 homoeologous gene in subgenomes A or B were extensively transcribed in 12 tissues, respectively, while the other copies were barely transcribed in 12 tissues.

Supplementary Figure 13. Expression patterns of *pdl1* and *acsl6* genes in 12 tissues in *C. carpio*.

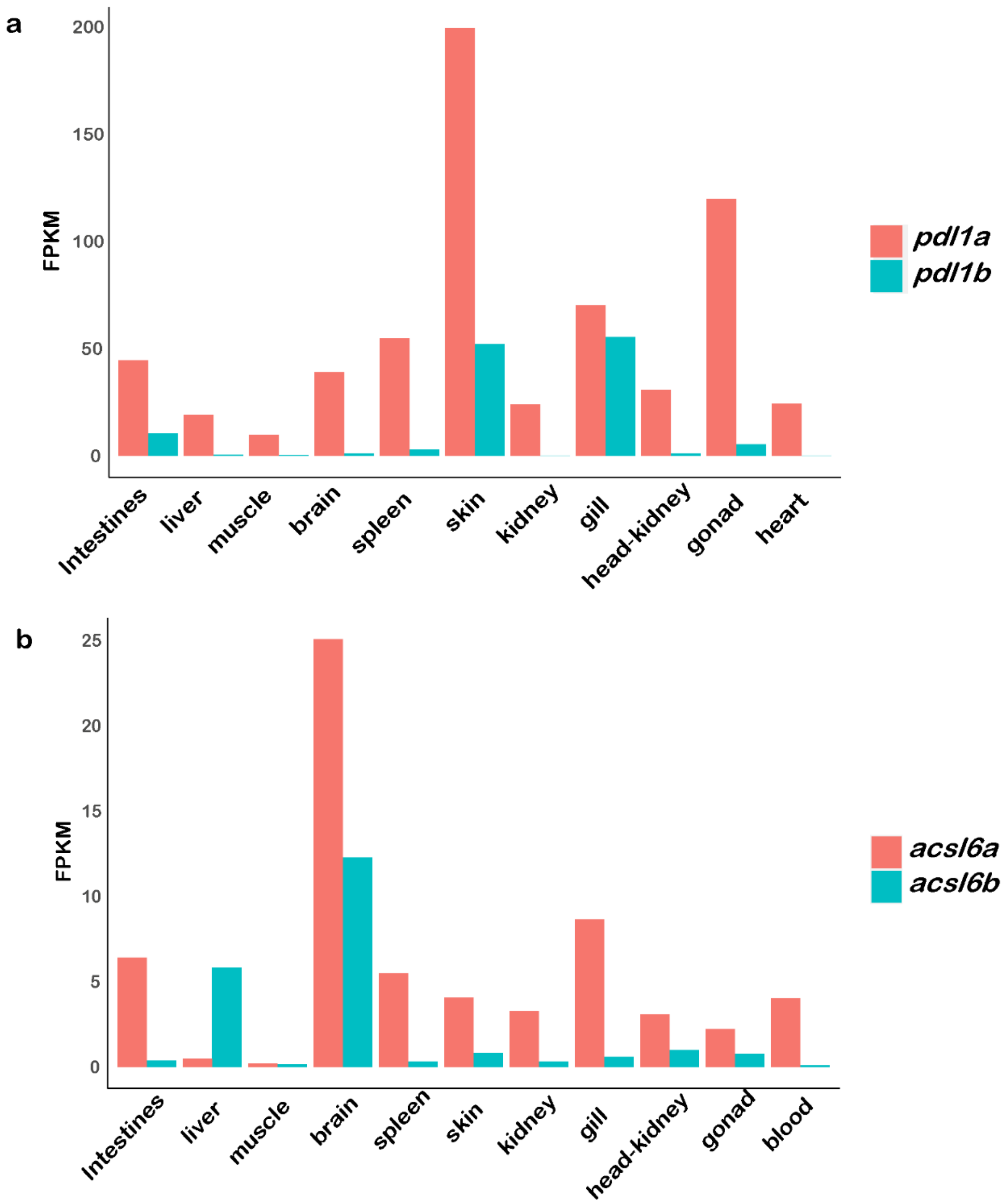

Note: **a**. Expression patterns of *pdl1* genes in 12 tissues; **b**. Expression patterns of *acsl6* genes in 12 tissues.

Supplementary Figure 14. Asymmetric homoeologous expression patterns under biotic or abiotic stresses in *C. carpio*.

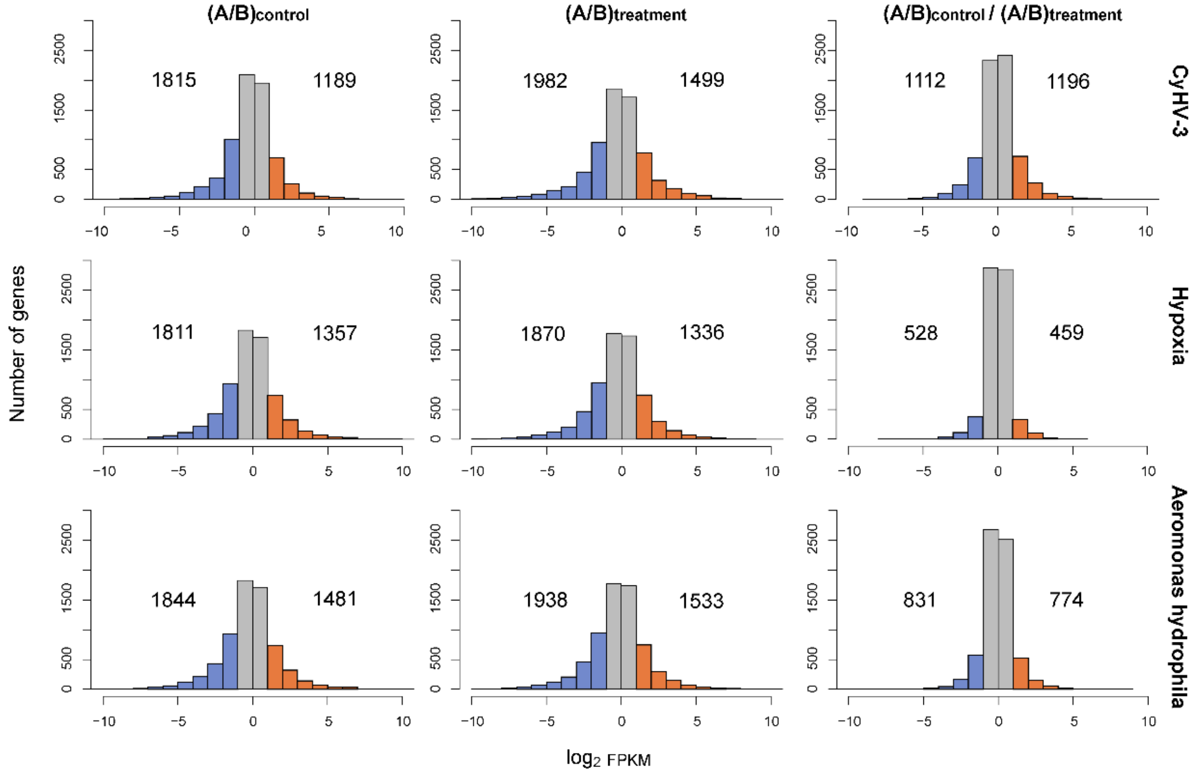

The RNA-seq data for biotic and abiotic stresses treatment were collected from SRA databases (Accession No. PRJNA314552 for CyHV-3 infection, Accession No. PRJNA315069 for *Aeromonas hydrophila* infection, and No. PRJNA512071 for hypoxia experiment).

Supplementary Figure 15. Methylation levels of asymmetrically expressed homoeologous genes in 12 tissues in *C. carpio*.

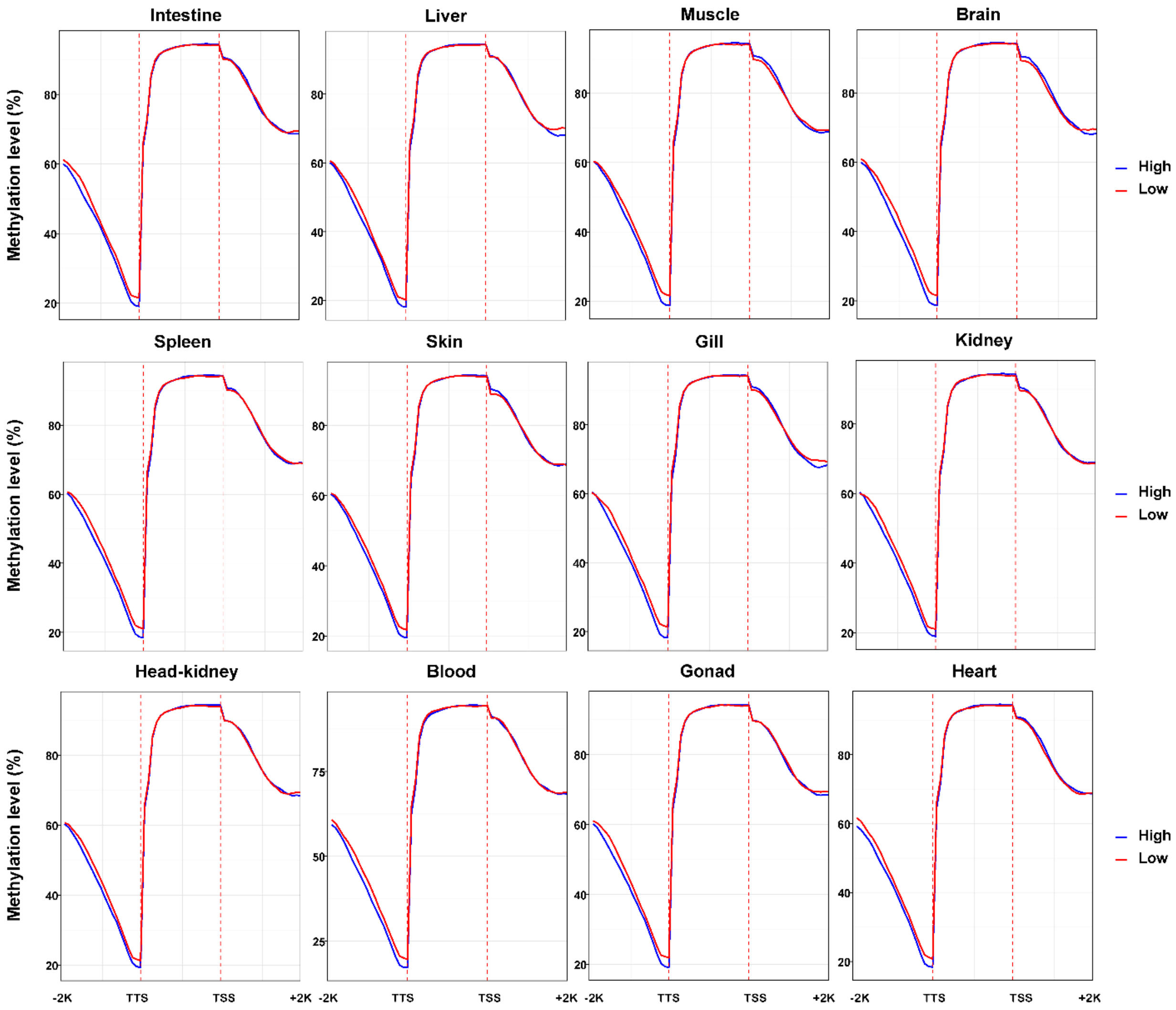

Note: High means highly expressed genes, low means lowly expressed genes.

Supplementary Figure 16. CG methylation levels of divergent expressed homoeologous genes in *C. carpio*.

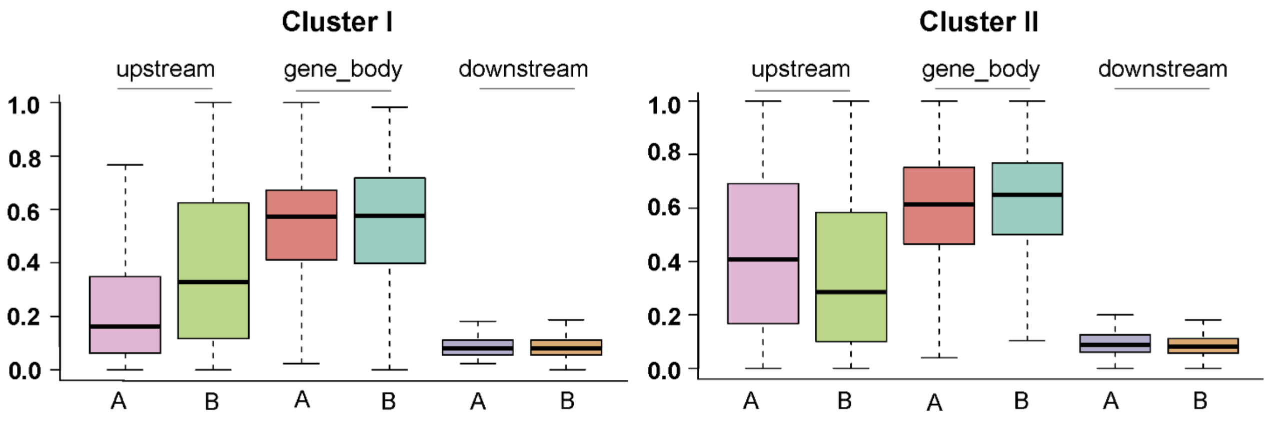

Note: Homoeologous genes in Cluster I were dominantly expressed in subgenome A, but silenced in subgenome B; homoeologous genes in Cluster II were dominantly expressed in subgenome B, but silenced in subgenome A.

Supplementary Tables

Supplementary Table 1. Summaries of library construction and sequencing data of three common carp strains.

Strain	Library insert	Total base (Gbp)	Read length (bp)	Sequence coverage (X)
Hebao red carp	250 bp	77.69	150	48.56
	350 bp	53.51	150	33.44
	500 bp	47.47	150	29.67
	2 Kb	27.96	150	17.48
	5 Kb	32.56	125	20.35
	10 Kb	46.22	125	28.89
	20 Kb	13.24	125	8.28
	Total	298.65		186.66
Yellow River carp	250 bp	89.88	150	56.18
	500 bp	77.01	150	48.13
	2 Kb	66.98	150	41.86
	5 Kb	38.35	150	23.96
	10 Kb	34.54	150	21.59
	15 Kb	18.41	150	11.51
	20 Kb	13.94	150	8.71
	Total	339.11		211.94
German mirror carp	250 bp	89.11	150	59.80
	500 bp	73.54	150	49.36
	2 Kb	58.85	150	39.50
	5 Kb	37.18	150	24.95
	10 Kb	35.69	150	23.96
	15 Kb	17.25	150	11.58
	20 Kb	18.77	150	12.59
	Total	330.39		221.74

Supplementary Table 2. The genome assembly statistics of three common carp strains.

Strains		Length (bp)		Number	
		Contig	Scaffold	Contig	Scaffold
Hebao red carp	Total	1,409,910,433	1,460,461,589	355,804	262,449
	Max	207,110	6,571,350	-	-
	>=2000 bp	-	-	87,986	9,135
	N50	20,684	923,370	19,142	393
	N60	15,917	629,080	26,914	589
	N70	11,609	420,074	37,264	873
	N80	7,300	205,075	52,431	1,362
	N90	2,325	22,238	84,081	3,290
Yellow River carp	Total	1,392,182,197	1,424,610,504	254,656	144,810
	Max	217,672	10,558,934	-	-
	>=2000 bp	-	-	89,155	8,761
	N50	21,811	1,706,244	18,518	211
	N60	17,102	1,268,502	25,724	308
	N70	12,799	878,940	35,132	444
	N80	8,734	424,730	48,213	674
	N90	4,302	97,382	70,189	1,331
German mirror carp	Total	1,359,861,568	1,415,500,317	76,948	18,344
	Max	409,856	14,180,042	-	-
	>=2000 bp	-	-	47,212	5,069
	N50	52,141	3,465,820	7,457	107
	N60	40,549	2,472,690	10,411	157
	N70	30,524	1,769,284	14,280	224
	N80	20,903	1,135,885	19,641	324
	N90	11,395	421,233	28,250	520

Supplementary Table 3. Summaries of genetic linkage maps of three common carp strains.

Species	SNP	Locus	Length (cM)	Average marker interval (cM)	Average locus interval (cM)
YR	28,194	14,146	10,596	0.38	0.75
HB	29,019	16,183	21,964	0.76	1.36
GM	32,160	23,936	19,425	0.60	0.81

Note: YR, Yellow River carp; HB, Hebao red carp; GM, German mirror carp.

Supplementary Table 4. Statistics of three integrated genome assemblies.

Species	Genome size (bp)	Anchored genome size (bp)	Contig N50 (bp)	Scaffold N50 (bp)	Gene content	Anchored gene content	Anchored genome ratio	Anchored gene ratio
YR	1,424,950,904	1,261,167,219	21,811	1,706,244	44626	42,303	88.51%	94.79%
HB	1,510,402,370	1,241,545,686	20,684	923,370	44269	41,569	82.20%	93.90%
GM	1,416,103,994	1,304,360,463	52,141	3,465,820	44758	43,577	92.11%	97.36%

Note: YR, Yellow River carp; HB, Hebao red carp; GM, German mirror carp.

Supplementary Table 5. Completeness assessment of three common carp genomes.

Species	Complete		Complete + partial	
	proteins	%completeness	proteins	%completeness
HB	216	87.10	243	97.98
YR	216	87.10	245	98.79
GM	215	86.69	245	98.79

Note: YR, Yellow River carp; HB, Hebao red carp; GM, German mirror carp.

Supplementary Table 6. Genic region coverage assessed based on ESTs in three common carp genome assemblies

Strain	Data set	Number	Total length (bp)	Sequences Covered by assembly (%)	With >90% sequences in one scaffold		With >50% sequences in one scaffold	
					Number	Percent (%)	Number	Percent (%)
HB	>0 bp	13,198	7,679,659	99.86	12,433	94.20	13,135	99.52
	>200 bp	13,198	7,679,659	99.86	12,433	94.20	13,135	99.52
	>500 bp	4,279	5,036,033	99.91	3,942	92.12	4,250	99.32
	>1 Kb	1,793	3,310,438	99.89	1,621	90.41	1,778	99.16
	>2 Kb	517	1,528,476	99.81	458	88.59	511	98.84
YR	>0 bp	23,352	12,465,030	99.26	21,647	92.70	22,996	98.48
	>200 bp	23,352	12,465,030	99.26	21,647	92,70	22,996	98.48
	>500 bp	7,285	7,714,476	99.64	6,829	93.74	7,225	99.18
	>1 Kb	2,764	4,589,503	99.89	2,601	94.10	2,749	99.46
	>2 Kb	583	1,590,318	99.83	547	93.83	577	98.97
GM	>0 bp	23,409	13,865,382	99.20	21,770	93.00	22,998	98.24
	>200 bp	23,409	13,865,382	99.20	21,770	93.00	22,998	98.24
	>500 bp	8,077	9,321,683	99.68	7,539	93.34	7,992	98.95
	>1 Kb	3,484	6,119,622	99.94	3,287	94.35	3,464	99.43
	>2 Kb	894	2,522,202	99.89	835	93.40	887	99.22

Supplementary Table 7. Transposable elements in common carp genomes.

Repeat class	Hebao red carp		Yellow River carp		German mirror carp	
	length	ratio (%)	length	ratio (%)	length	ratio (%)
DNA/hAT	46,187,953	3.06	42,708,696	2.99	39,955,117	2.82
DNA/TcMar-Tc1	76,687,355	5.08	71,927,799	5.05	74,796,224	5.28
DNA/other	86,767,046	5.74	84,504,475	5.93	78,029,897	5.51
LINE/L2	64,392,604	4.26	63,835,670	4.48	60,937,527	4.31
LINE/other	45,027,580	2.98	41,679,366	2.92	39,509,774	2.79
LTR/Gypsy	40,777,238	2.69	36,324,585	2.55	36,049,641	2.55
LTR/other	66,297,715	4.39	59,795,318	4.19	58,439,616	4.13
RC/Helitron	14,231,312	0.94	13,432,925	0.94	13,134,833	0.93
Unknown	66,929,472	4.43	54,729,884	3.84	47,539,485	3.36
Other	50,576,220	3.34	50,043,491	3.51	45,707,911	3.22
Total	557,874,495	36.94	518,982,209	36.42	494,100,025	34.89

Supplementary Table 8. Gene prediction in common carp genome.

Species	Genome length (bp)	Total gene length (bp)	Gene content	Exon content	Exon number per gene	Gene density	Average gene length (bp)
YR	1,424,950,904	616,471,225	44,626	426,870	9.57	0.43	13,814.17
HB	1,510,402,370	602,203,106	44,269	418,078	9.44	0.40	13,603.27
GM	1,416,103,994	626,902,020	44,758	428,776	9.58	0.44	14,006.48

Supplementary Table 9. The statistics of gene structure of *C. carpio* and the comparison with other teleosts.

Gene Set	Genome assembly size (Mb)	No. genes	Mean CDS length	No. exons per gene	Mean exon size (bp)	Mean intron size (bp)
YR	1,425	44,626	1,648	9.57	172	1,420
HB	1,510	44,269	1,641	9.44	174	1,417
GM	1,416	44,758	1,650	9.58	172	1,440
D. rerio	1,412	26,163	1,853	7.97	232	2,923
C. idellus	900	27,263	1,539	8.50	250	1,287
G.aculeatus	461	20,787	1,592	9.88	161	771
O.latipes	868	19,686	1,553	10.04	154	1,215
T.rubripes	393	18,523	1,617	10.69	151	600

Note: YR: Yellow River carp, HB: Hebao red carp, GM: German mirror carp.

Supplementary Table 10. Gene annotation in common carp genome.

Database	Hebao red carp	Yellow River carp	German mirror carp
Swiss-prot	41,019<br>(92.6%)	40,710<br>(91.2%)	40,790<br>(91.1%)
NR	42,893<br>(96.9%)	42,909<br>(96.2%)	43,016<br>(96.1%)
KEGG	37,258<br>(84.1%)	37,108<br>(83.2%)	37,210<br>(83.1%)
GO	31,004<br>(70%)	31,041<br>(69.6%)	31,154<br>(69.6%)
Pfam	37,713<br>(85.2%)	37,797<br>(84.7%)	37,859<br>(84.6%)
InterPro	40,872<br>(92.3%)	40,721<br>(91.2%)	40,772<br>(91.1%)
Annotated	42,916<br>(96.9%)	42,916<br>(96.2%)	43,055<br>(96.2%)

Supplementary Table 11. Accession numbers of *rag2* genes used in this study.

Species	Gene	Accession Number
Acrossocheilus hemispinus	*rag2*	DQ366986
Onychostoma barbatula	*rag2*	DQ366964
Onychostoma angustistomata	*rag2*	HQ235896
Hampala macrolepidota	*rag2*	DQ366965
Puntius tetrazoma	*rag2*	DQ366938
Puntius sophore	*rag2*	HQ235899
Mystacoleucus marginatus)	*rag2*	HQ235894
Poropuntius huangchuchieni	*rag2*	DQ366952
Sinocyclocheilus anshuiensis	*rag2a*	XM_016249750.1
Sinocyclocheilus anshuiensis	*rag2b*	XM_016466752.1
Garra tana	*rag2*	HQ235879
Cirrhinus molitorella	*rag2*	DQ366959
Discogobio laticeps	*rag2*	DQ366949
Sinilabeo laticeps	*rag2*	HQ235885
Ctenopharyngodon idella	*rag2*	DQ366996
Danio apogon	*rag2*	U71094

Supplementary Table 12. Genome sequencing and assembly of diploid species.

Species	Raw reads	Raw Bases	Clean reads	Clean Bases
Poropuntius huangchuchieni	191,169,831	57,350,949,300	163,806,576	49,141,972,800
Cirrhinus molitorella	182,277,569	54,683,270,700	156,250,025	46,875,007,500
Onychostoma barbatula	214,371,286	64,311,385,800	183,276,646	54,982,993,800
Hampala macrolepidota	479,970,766	71,995,614,900	478,507,722	71,776,158,300

Species		Length (bp)		Number	
		Contig	Scaffold	Contig	Scaffold
Poropuntius huangchuchieni	Total	846,673,898	868,595,308	1,667,071	1,431,194
	Max	76,402	140,771	-	-
	Number>=2000	-	-	82,623	84,023
	N50	1,574	2,176	109,206	76,628
Cirrhinus molitorella	Total	953,783,459	985,712,601	1,271,496	976,377
	Max	93,971	145,483	-	-
	Number>=2000	-	-	139,665	139,179
	N50	2,444	3,824	109,206	76,628

Supplementary Table 13. Chromosome nomenclature of *C. carpio* according to *D. rerio*.

D. rerio	*C. carpio*	
	subgenome A	subgenome B
LG1	A01	B01
LG2	A02	B02
LG3	A03	B03
LG4	A04	B04
LG5	A05	B05
LG6	A06	B06
LG7	A07	B07
LG8	A08	B08
LG9	A09	B09
LG10	A10	B10
LG11	A11	B11
LG12	A12	B12
LG13	A13	B13
LG14	A14	B14
LG15	A15	B15
LG16	A16	B16
LG17	A17	B17
LG18	A18	B18
LG19	A19	B19
LG20	A20	B20
LG21	A21	B21
LG22	A22	B22
LG23	A23	B23
LG24	A24	B24
LG25	A25	B25

Note: Chromosomes in *C. carpio* were named as A and B based on the progenitor genome.

Supplementary Table 14. Gene present and loss categories based on distinguished A/B subgenome.

Category	Content	Subgenome
CID:CCA:CCB=1:0:1	1220	B
CID:CCA:CCB=1:1:0	915	A
CID:CCA:CCB=1:0:2 total	110	B
CID:CCA:CCB=1:0:2 oneChr	88	B
CID:CCA:CCB=1:0:2 twoChr	22	B
CID:CCA:CCB=1:2:0 total	126	A
CID:CCA:CCB=1:2:0 oneChr	102	A
CID:CCA:CCB=1:2:0 twoChr	24	A
CID:CCA:CCB=1:1:1 total	8353	A & B
CID:CCA:CCB=1:1:1 paired	8291	A & B
CID:CCA:CCB=1:1:1 non-paired	62	A & B

Note: CID, *Ctenopharyngodon idella*; CCA, A subgenome of *C. carpio*; CCB, *C. carpio* B subgenome of *C. carpio*.

Supplementary Table 15. Validation of homoeologous rearrangement using mate-paired BAC-end sequences.

Total BAC reads	BAC pair mapped	Homo pair mapped	Rearrangement mapped	Confirmed rearrangement	Total rearrangement
34,932	26,350	3,269	96	35	92
	75.43%	9.36%	0.27%	38.04%	

We collected a total of 34,932 mate-paired BAC-end sequences (BES) (GenBank accession numbers of HN150714-HN153235 and HR505563-HR575920) and mapped 26,350 BES pairs onto 50 chromosomes of common carp. Of the mapped 26,350 BES pairs, only 3,269 BES pairs were successfully mapped onto two homoeologous chromosomes simultaneously, indicating that two subgenomes are highly divergent with relatively low sequence similarity. We therefore used these 3,269 BES pairs to validate homoeologous chromosomal rearrangement. We identified 96 BES pairs that mapped on the breakpoints of 35 rearrangement regions between two subgenomes, accounting to 38.04% of the identified 92 rearrangement regions. However, we still could not say that the remaining 57 rearrangement regions were due to assembly errors because the sequence similarity between two subgenomes is too low, as shown by the low homoeologous mapping ratio, and the BES pairs may not be able to map onto the breakpoints of rearrangement regions in two homoeologous chromosomes simultaneously.

Supplementary Table 16. Transcriptome data from 12 tissues of *C. carpio*.

Tissue	Raw reads	Clean reads	Clean bases (Gbp)	Total mapped reads	Q20 (%)	Q30 (%)	GC content (%)
Intestine	58,594,984	57,016,106	8.55	48,197,574 (84.53%)	96.60	91.48	45.81
Liver	50,842,422	40,228,218	6.03	35,166,850 (87.42%)	96.99	92.20	45.92
Muscle	46,531,258	36,744,052	5.51	32,616,748 (88.77%)	96.80	91.72	47.79
Brain	53,510,636	42,260,310	6.34	36,590,143 (86.58%)	96.53	91.26	44.12
Spleen	49,081,138	38,553,610	5.78	32,503,886 (84.31%)	96.01	90.22	45.27
Skin	55,044,348	43,528,204	6.53	36,769,595 (84.47%)	96.66	91.62	45.17
Gill	47,026,858	37,045,818	5.56	31,717,259 (85.62%)	96.77	91.75	44.74
Kidney	48,014,150	37,875,804	5.68	31,945,848 (84.34%)	96.61	91.36	46.65
Head-kidney	44,363,242	35,042,086	5.26	29,912,694 (85.36%)	96.57	91.28	47.51
Blood	38,771,190	37,779,062	5.67	31,642,491 (83.76%)	95.08	87.70	45.40
Gonad	44,232,212	34,908,582	5.24	29,941,006 (85.77%)	96.43	91.13	46.92
Heart	51,011,754	40,348,926	6.05	35,910,413 (89.00%)	96.62	91.35	45.72

Supplementary Table 17. Homoeologous gene expression divergence in two subgenomes.

Tissues	log2(A/B（FPKM）)									
	1	-1	2	-2	3	-3	4	-4	5	-5
Intestine	1255	1764	534	837	273	444	147	248	82	148
Liver	1342	1647	574	774	271	375	132	199	65	104
Muscle	1439	1977	577	835	240	341	96	168	46	77
Brain	1258	1651	447	666	207	308	99	152	53	76
Spleen	1277	1696	560	769	274	404	146	202	82	106
Skin	1336	1800	469	650	178	247	74	87	27	34
Gill	1285	1822	504	809	231	416	119	216	56	110
Kidney	1539	1853	682	852	322	432	165	236	91	130
Head kidney	1613	1945	656	886	281	425	136	223	63	103
Blood	1372	1643	691	823	375	449	210	257	107	145
Gonad	1441	1862	557	822	239	426	114	221	53	113
Heart	1350	1773	547	819	268	408	130	225	69	112
12 tissues	4719	5403	2590	3225	1349	1802	728	1067	406	627
	62.62%	71.70%	51.33%	63.91%	45.56%	60.86%	41.82%	61.29%	39.88%	61.59%
One-way	2133	2817	1821	2456	1159	1612	674	1013	391	612
	28.30%	37.38%	36.09%	48.67%	39.14%	54.44%	38.71%	58.18%	38.41%	60.12%
Swing	2586		769		190		54		15	
	34.32%		15.24%		6.42%		3.10%		1.47%	
Overall	7536		5046		2961		1741		1018	
	91.00%		60.93%		35.76%		21.02%		12.29%	

Note: 8077 of the 8291 homoeologous gene pairs were expressed in the 12 tissues.

Supplementary Table 18. Transcriptome data from 12 tissues of *C. idella*.

Tissue	Raw reads	Clean reads	Clean bases (Gbp)	Total mapped reads	Q20 (%)	Q30 (%)	GC content (%)
Intestine	43,258,982	41,811,030	6.27	37,306,691 (89.23%)	96.92	92.22	43.46
Liver	42,896,884	41,453,584	6.22	37,858,359 (91.33%)	96.98	92.24	45.29
Muscle	44,991,472	43,592,278	6.54	40,341,350 (92.54%)	96.98	92.23	46.75
Brain	54,535,354	43,120,570	6.47	39,382,263 (91.33%)	96.73	91.72	43.69
Spleen	55,518,368	43,834,564	6.58	39,105,328 (89.21%)	96.59	91.44	43.82
Skin	48,191,282	38,073,406	5.71	34,318,332 (90.14%)	96.75	91.71	45.59
Gill	48,740,776	38,515,054	5.78	34,820,462 (90.41%)	96.66	91.56	44.01
Kidney	52,425,680	41,487,996	6.22	37,389,070 (90.12%)	96.80	91.86	43.92
Head-kidney	47,771,592	37,711,358	5.66	33,816,587 (89.67%)	96.63	91.48	44.23
Blood	58,843,694	57,318,250	8.60	51,253,377 (89.42%)	96.93	92.15	45.32
Gonad	46,647,678	36,735,846	5.51	32,338,397 (88.03%)	96.41	91.22	46.24
Heart	50,878,226	40,240,458	6.04	37,180,686 (92.40%)	96.79	91.74	44.77

Supplementary Table 19. Homoeologous gene expression under abiotic and disease stresses.

Stress	Category	Description	No. of gene pairs
Hypoxia	Expressed gene pairs	FPKM>0	6,708
	Treatment	A dominant	1,336
		B dominant	1,870
		balanced	3,502
	Control	A dominant	1,357
		B dominant	1,811
		balanced	3,540
	(A/B)treatment / (A/B)control	A changed faster	459
		B changed faster	528
		balanced	5,721
CyHV-3	Expressed gene pairs	FPKM>0	7,054
	Treatment	A dominant	1,499
		B dominant	1,982
		balanced	3,573
	Control	A dominant	1,189
		B dominant	1,815
		balanced	4,050
	(A/B)treatment / (A/B)control	A changed faster	1,196
		B changed faster	1,112
		balanced	4,746
Aeromonas	Expressed gene pairs	FPKM>0	6,802
hydrophila	Treatment	A dominant	1,533
		B dominant	1,938
		balanced	3,331
	Control	A dominant	1,481
		B dominant	1,844
		balanced	3,478
	(A/B)treatment / (A/B)control	A changed faster	774
		B changed faster	831
		balanced	5,197

Note: Homoeologous pairs had (A/B) expression values less than 2-fold were regarded as balanced.

Supplementary Table 20. Differentially expressed genes of 8291 homoeologous gene pairs under abiotic and biotic stresses.

Stress	Category	No. of genes	Ratio_1	Total_1	Ratio_2	Total
Hypoxia	No response	10,980	76.53%			
	Up regulated	479	3.34%	3,367	23.47%	14,347
	Down regulated	2,888	20.13%			
CyHV-3	No response	9,137	61.30%			
	Up regulated	1,584	10.63%	5,768	38.70%	14,905
	Down regulated	4,184	28.07%			
Aeromonas hydrophila	No response	11,120	76.33%			
	Up regulated	1,533	10.52%	3,448	23.67%	14,568
	Down regulated	1,915	13.15%			

Note: Homoeologous pairs had Treatment /Control expression values less than 2-fold were regarded as no response.

Supplementary Table 21. Differentially expressed homoeologous gene pairs of the 8291 homoeologous gene pairs under abiotic and biotic stresses.

Stress	Category	No. of gene pairs	Ratio_1	Total_1	Ratio_2	Total
Hypoxia	No response	5,553	82.78%	1,155	17.22%	6,708
	Up regulated	126	1.88%			
	Down regulated	1,029	15.34%			
CyHV-3	No response	4,757	67.44%	2,297	32.56%	7,054
	Up regulated	613	8.69%			
	Down regulated	1,684	23.87%			
Aeromonas hydrophila	No response	5,615	82.55%	1,187	17.45%	6,802
	Up regulated	607	8.92%			
	Down regulated	580	8.53%			

Note: Homoeologous pairs had Treatment /Control expression values less than 2-fold were regarded as no response.

Supplementary Table 22. Whole genome methylation data of common carp.

Samples	Clean_reads	Clean_bases	Q20(%	Q30(%)
01	224,926,131	67.48	96.19	90.15
02	245,729,148	73.72	96.49	90.78
03	240,069,205	72.02	96.31	90.42

Supplementary Table 23. Whole genome methylation levels of common carp.

Categories	C	CG	CHG	CHH	mC	mCG	mCHG	mCHH	mC/C	mCG/CG
Whole_genome	309,953,955	24,691,159	68,492,608	216,770,188	21,724,824	21,275,948	79,756	369,120	7.01%	86.17%
Subgenome_A	150,548,530	11,808,242	33,367,121	105,373,167	10,363,257	10,152,778	37,623	172,856	6.88%	85.98%
Subgenome_B	159,405,425	12,882,917	35,125,487	111,397,021	11,361,567	11,123,170	42,133	196,264	7.13%	86.34%

Supplementary References

1. Peng, W. *et al.* An ultra-high density linkage map and QTL mapping for sex and growth-related traits of common carp (Cyprinus carpio). *Scientific Reports* **6**, 26693 (2016).
2. Feng, H., Conneely, K.N. & Wu, H. A Bayesian hierarchical model to detect differentially methylated loci from single nucleotide resolution sequencing data. *Nucleic acids research* **42**, e69-e69 (2014).
