# Supplementary material for: The allotetraploid origin and asymmetrical genome evolution of the common carp Cyprinus carpio
Source: Nat Commun. 2019 Oct 11;10:4625. doi: 10.1038/s41467-019-12644-1 (PMC6789147; doi:10.1038/s41467-019-12644-1)
Supplement: Supplementary file 3 — Description of Additional Supplementary Files [file 41467_2019_12644_MOESM3_ESM.pdf]

Description of Additional Supplementary Files

File Name: Supplementary Data 1.
Description: Subgenome summary of *C. carpio*.

File Name: Supplementary Data 2.
Description: Annotation, GO and KEGG analysis of single copy genes in two subgenomes.

File Name: Supplementary Data 3.
Description: Chromosome translocated genes in subgenomes.

File Name: Supplementary Data 4.
Description: Confirmed rearrangement regions using mate-paired BAC-end sequences.

File Name: Supplementary Data 5.
Description: Selective pressure of two subgenomes.

File Name: Supplementary Data 6.
Description: Gene list of the 8291 homoeologous gene pairs.

File Name: Supplementary Data 7.
Description: Expression levels of the 8291 homoeologous gene pairs.

File Name: Supplementary Data 8.
Description: Annotation of the divergent genes in 12 tissues in *C. carpio*.

File Name: Supplementary Data 9.
Description: Annotation, GO and KEGG analysis of the 32 fold-change genes between two subgenomes of the 12 tissues in *C. carpio*.

File Name: Supplementary Data 10.
Description: Genes in the three clusters from the comparison between common carp and grass carp.

File Name: Supplementary Data 11.
Description: Annotation of the extremely divergent expressed homoeologous genes.

File Name: Supplementary Data 12.
Description: GO and KEGG analysis of the extremely divergent expressed homoeologous genes.
