# Supplementary material for: The allotetraploid origin and asymmetrical genome evolution of the common carp Cyprinus carpio
Source: Nat Commun. 2019 Oct 11;10:4625. doi: 10.1038/s41467-019-12644-1 (PMC6789147; doi:10.1038/s41467-019-12644-1)
Supplement: Supplementary file 16 — Reporting Summary [file 41467_2019_12644_MOESM16_ESM.pdf]

nature research

Corresponding author(s): DBPR Last

updated by author(s): Sept. 16, 2019

Reporting Summary

Nature Research wishes to improve the reproducibility of the work that we publish. This form provides structure for consistency and transparency in reporting. For further information on Nature Research policies, see Authors & Referees and the Editorial Policy Checklist.

Statistics

For all statistical analyses, confirm that the following items are present in the figure legend, table legend, main text, or Methods section.

n/a	Confirmed	
☐	☒	The exact sample size (n) for each experimental group/condition, given as a discrete number and unit of measurement
☐	☒	A statement on whether measurements were taken from distinct samples or whether the same sample was measured repeatedly
☐	☒	The statistical test(s) used AND whether they are one- or two-sided<br>*Only common tests should be described solely by name; describe more complex techniques in the Methods section.*
☐	☒	A description of all covariates tested
☐	☒	A description of any assumptions or corrections, such as tests of normality and adjustment for multiple comparisons
☐	☒	A full description of the statistical parameters including central tendency (e.g. means) or other basic estimates (e.g. regression coefficient) AND variation (e.g. standard deviation) or associated estimates of uncertainty (e.g. confidence intervals)
☐	☒	For null hypothesis testing, the test statistic (e.g. F, t, r) with confidence intervals, effect sizes, degrees of freedom and P value noted<br>*Give P values as exact values whenever suitable.*
☒	☐	For Bayesian analysis, information on the choice of priors and Markov chain Monte Carlo settings
☒	☐	For hierarchical and complex designs, identification of the appropriate level for tests and full reporting of outcomes
☐	☒	Estimates of effect sizes (e.g. Cohen's d, Pearson's r), indicating how they were calculated

Our web collection on statistics for biologists contains articles on many of the points above.

Software and code

Policy information about availability of computer code

Data collection	Genome sequences of diploid species and RNA-seq data under stresses were download from NCBI SRA using FTP or Aspera.
Data analysis	Data were analyzed using R 3.5.1. Other analysis tools used include ClustalW, MEGA7, OrthoMCL, Hisat2, PAML, Bismark. Details found in methods section.

For manuscripts utilizing custom algorithms or software that are central to the research but not yet described in published literature, software must be made available to editors/reviewers. We strongly encourage code deposition in a community repository (e.g. GitHub). See the Nature Research guidelines for submitting code & software for further information.

Data

Policy information about availability of data

All manuscripts must include a data availability statement. This statement should provide the following information, where applicable:

- Accession codes, unique identifiers, or web links for publicly available datasets
- A list of figures that have associated raw data
- A description of any restrictions on data availability

All the genomic sequence datasets have been deposited in the NCBI Sequence Read Archive under accession number PRJNA510861, PRJNA511029-PRJNA511032.

Field-specific reporting

Please select the one below that is the best fit for your research. If you are not sure, read the appropriate sections before making your selection.

☒ Life sciences ☐ Behavioural & social sciences ☐ Ecological, evolutionary & environmental sciences

Life sciences study design

All studies must disclose on these points even when the disclosure is negative.

Sample size	One individual sample is used in whole genome sequencing. For whole genome resequencing, 16 samples were used and three samples were used in bisulfite sequencing and RNA-seq.
Data exclusions	Genes with low read counts (FPKM value <1) were excluded from the analysis.
Replication	All replications were successful. The experimental findings are supported by the biological replicates.
Randomization	Samples for genome sequencing, resequencing, bisulfite sequencing and RNA-seq were collected randomly form healthy adult fishes.
Blinding	N/A

Reporting for specific materials, systems and methods

We require information from authors about some types of materials, experimental systems and methods used in many studies. Here, indicate whether each material, system or method listed is relevant to your study. If you are not sure if a list item applies to your research, read the appropriate section before selecting a response.

Materials & experimental systems

n/a	Involved in the study	
☒	☐	Antibodies
☒	☐	Eukaryotic cell lines
☒	☐	Palaeontology
☐	☒	Animals and other organisms
☒	☐	Human research participants
☒	☐	Clinical data

Methods

n/a	Involved in the study	
☒	☐	ChIP-seq
☒	☐	Flow cytometry
☒	☐	MRI-based neuroimaging

Animals and other organisms

Policy information about studies involving animals; ARRIVE guidelines recommended for reporting animal research

Laboratory animals	Female individuals of the three common carp strains were used for genome sequencing, namely Hebao red carp, Yellow River carp and German mirror carp. Diploid species used for genome sequencing are healthy adult.
Wild animals	N/A
Field-collected samples	N/A
Ethics oversight	This study was carried out in accordance with the recommendations of the care and use of animals for scientific purposes set up by the Animal Care and Use Committee of Chinese Academy of Fishery Sciences (ACUC-CAFS).

Note that full information on the approval of the study protocol must also be provided in the manuscript.
